# Meta-Analysis of Peripheral Blood Transcriptome Datasets Reveals a Biomarker Panel for Tuberculosis in Patients Infected With HIV

**DOI:** 10.3389/fcimb.2021.585919

**Published:** 2021-03-19

**Authors:** Yahong Chen, Qiaowen Wang, Shujin Lin, Jinglan Lai, Jing Lin, Wen Ao, Xiao Han, Hanhui Ye

**Affiliations:** ^1^ Mengchao Hepatobiliary Hospital of Fujian Medical University, Fuzhou, China; ^2^ College of Biological Science and Engineering, Fuzhou University, Fuzhou, China

**Keywords:** human immunodeficiency virus, tuberculosis, transcriptome, microarray, meta-analysis

## Abstract

Biomarkers are critical for rapid diagnosis of tuberculosis (TB) and could benefit patients with AIDS where diagnosis of TB co-infection is challenging. Meta-analysis is an approach to combine the results of the studies with standard statistical method by weighting each study with different sample size. This study aimed to use meta-analysis to integrate transcriptome datasets from different studies and screen for TB biomarkers in patients who were HIV-positive. Five datasets were subjected to meta-analysis on whole-blood transcriptomes from 640 patients infected with HIV. A total of 293 differentially expressed genes (DEGs) were identified as significant (P<0.0001) using the random effective model to integrate the statistical results from each study. DEGs were enriched in biological processes related to TB, such as “Type I interferon signaling” and “stimulatory C-type lectin receptor signaling”. Eighteen DEGs had at least a two-fold change in expression between patients infected with HIV who were TB-positive and those who were TB-negative. *GBP4*, *SERPING1*, *ATF3* and *CDKBN3* were selected as a biomarker panel to perform multivariable logistic regression analysis on TB status and relative gene expression levels. The biomarker panel showed excellent accuracy (AUC>0.90 for HIV+TB) in clinical trial and suggests that meta-analysis is an efficient method to integrate transcriptome datasets from different studies.

## Introduction

Human immunodeficiency virus (HIV) and *Mycobacterium tuberculosis* (*M. tuberculosis*) are serious infectious diseases that are among the ten leading causes of death worldwide (Bell and Noursadeghi, 2018). Co-infection with HIV and *M. tuberculosis* aggravates the incidence of mortality. Infection with HIV greatly increases the risk of tuberculosis (TB) due to functional disruption of the immune response and loss of ability of the host to defend against *M. tuberculosis* infection (Amelio et al., 2019). Meanwhile, TB exacerbates progression of acquired immune deficiency syndrome (AIDS) *via* increasing levels of HIV replication, propagation and genetic diversity (Souriant et al., 2019). For patients with AIDS, TB is the predominant cause of death, accounting for almost 40% of HIV-related deaths (Gupta et al., 2015). In Africa, the prevalence of TB in patients infected with HIV has been increasing over the past 20 years (Cohen et al., 2010; Cox et al., 2012; Wong et al., 2012). Moreover, diagnosis of TB including sputum-based technique and TB DNA based method is challenging. The atypical presentation of HIV-associated TB restricts the sensitivity and specificity of the current sputum-based diagnosis technique in patients with HIV co-infection. Furthermore, false negatives in such tests are likely to be lethal to patients with HIV co-infections. A systematic review on global autopsy studies revealed that TB remains undiagnosed at the time of death in approximately 50% of patients who are HIV-positive (Gupta et al., 2015). Therefore, development of novel diagnostic and therapeutic tools for TB in HIV co-infections is crucial in tackling the global burden of these infectious diseases.

The development of high-throughput transcriptome approaches has enabled the identification of genes whose transcription discriminates between diseases. Host transcriptional profiling has been used to search for biomarkers in pathogen infections (Berry et al., 2010; Blankley et al., 2014; Burel and Peters, 2018). The first complete description of the human blood transcriptional signature of TB identified 393 transcript signatures for active TB and a specific 86-transcript signature that discriminates active TB from other inflammatory and infectious diseases (Berry et al., 2010). Another 16−gene signature in whole blood was reported to predict the risk of active disease in individuals with latent TB (Zak et al., 2016). These signatures could be potential diagnostic and therapeutic targets. Using genome-wide analysis of RNA expression, researchers have identified hundreds of characteristic genes and biological pathways for TB in the blood of patients infected with HIV (Anderson et al., 2014; Dawany et al., 2014). However, the acquired DEGs only partially overlapped. The results of multiple studies often have poor reproducibility for numerous reasons including limited sample sizes, experimental conditions, physiological conditions, heterogeneous pathology and genetic background (Zhang et al., 2008). Therefore, the reliability of each study is uncertain and, more importantly, some DEGs with small responses will be missed in individual studies. Minor changes in gene expression can have significant implications if the responses are highly consistent across multiple experiments (Choi et al., 2003). Recently, meta‐analysis approaches have been widely applied as a powerful tool to overcome the above limitations (Tseng et al., 2012).

Meta-analysis studies can integrate data from multiple individual studies and identify the candidate genes that are differentially expressed consistently across studies (Adams et al., 2008). It was used to study infection with West Nile virus (WNV) by comparing new lists of genes that were differentially expressed between WNV-infected neurological tissues and control samples (Kosch et al., 2018). Meta-analysis can also be very helpful for studying functional annotation among multi-gene families as shown with transcriptomic data for liver and heart samples from rats and mice (Reyes et al., 2017). Another study utilized integrative meta-analysis of expression data to identify various expressed genes (Zhang et al., 2017). However, their methods were not standard meta-analysis which just intend to average the P-value without consider the sample size from different studies.

In the present work, meta-analysis of five transcriptomic studies was conducted using microarray gene expression data to identify DEGs involved in responses to *M. tuberculosis* in patients who were HIV-positive. In addition, functional interpretation (gene ontology analysis and pathway analysis) of the DEGs identified in the meta-analysis was performed. These TB-specific changes may provide new insights into the response mechanism of HIV co-infection with *M. tuberculosis*, which in turn could generate novel diagnostic and therapeutic targets for TB in patients who are HIV-positive.

## Materials and Methods

### Subjects

Subjects were classified according to HIV and TB status and compared with a bio independent sample set in case-control design at the discovery and validation stage. The sample size was dependent on the availability of serum samples. Subjects were registered in a public hospital in Fuzhou, Fujian Province, China from 2007 to 2011. The subjects were HIV-positive and included symptomatic patients with tuberculosis and non-tuberculosis patients. Symptomatic patients were continuously registered and represented the spectrum of respiratory diseases to be tested for tuberculosis diagnosis in the clinical environment. The reference standard for clinical diagnosis of pulmonary tuberculosis was: patients with pulmonary tuberculosis have a positive sputum culture in any of the first three sputum samples, or have histological examination and/or negative culture by positive culture and/or nucleic acid amplification test and/or at another sputum point other than the first three sputum points (defined as the first three sputum points under the microscope FB negative, Mycobacterium negative). Cases diagnosed as culture-negative TB usually have TB risk factors. Extensive diagnosis excludes other bacterial, fungal or parasitic diseases, and does not respond to empirical antimicrobial treatment. The diagnosis of culture-negative tuberculosis is based on the criteria of the American Thoracic Society and the Centers for Disease Control and Prevention (CDC), and includes chest CT findings such as small or nodular abnormalities consistent with tuberculosis.

### Collection of Microarray Data

The Gene Expression Omnibus (GEO) database from the National Center for Biotechnology Information (www.ncbi.nlm.gov/geo/) was searched using the keyword “HIV” and limiting the research type to “expression profiling by array”.

Inclusion criteria for the dataset were: (i) the dataset must be genome-wide mRNA-expression chip data supported by the literature; (ii) the original or standardized dataset must be considered; (iii) each dataset must include >3 samples; (iv) the sample source should be peripheral blood cell from patients or cell line.

After these searches, gene-expression microarray data were obtained from the GEO database. Simultaneously, a corresponding control group was established, from which four independent microarray datasets with raw data were selected.

### Meta-Analysis of Differential Gene Expression

R language (R Center for Statistical Computing, Vienna, Austria) was used to process the data, conduct statistical analyses, and obtain the path through which the data changed together. A meta-analysis was conducted on the results with the random-effects model to obtain the combined differential expression of genes, statistical tests, and to input the genes into the Database for Annotation, Visualization and Integrated Discovery [DAVID; http://david.abcc.ncifcrf.gov/ (Huang da et al., 2009)] to obtain the possible enriched pathway.

### Gene Enrichment Using the Gene Ontology and Kyoto Encyclopedia of Genes and Genomes Databases and Disease Analysis

DEGs were defined through functional interpretation using DAVID. Statistical analyses were performed, with p<0.05 denoting significance. A gene-symbol list was obtained and uploaded into DAVID. The GO and KEGG databases were also used. To unify the format of the DEGs, a functional annotation chart was used for uploading and analyses.

### RNA Extraction and Quantitative Reverse Transcription-Polymerase Chain Reaction

Total RNA was extracted using an RNA Isolation kit according to the manufacturer’s instructions. A NanoDrop™ ND-1000 spectrophotometer (Thermo Fisher Scientific, Wilmington, MA, USA) was used to determine the quantity and purity of RNA. Expression of complementary DNA was measured using a Prime Script™ RT kit (TaKaRa Biotechnology, Otsu, Japan). qPCR was undertaken on a 7900HT fast RT qPCR instrument (Applied Biosystems, Foster City, CA, USA) according to manufacturer’s instructions. Actin RNA was used as an internal control. The expression level was normalized to the average value in the control group to obtain the relative level.

### Statistics and Models

Clinical prediction models yielded two equations that express the probability of TB as a function of the statistically significant variables identified by multivariable logistic regression. The prediction models were tested with data from the validation set, and receiver-operating characteristic (ROC) curves were constructed. Models were evaluated by comparing the areas under the ROC curve in the derivation and validation sets.

## Results

### General Description of Datasets

**Figure 1:**
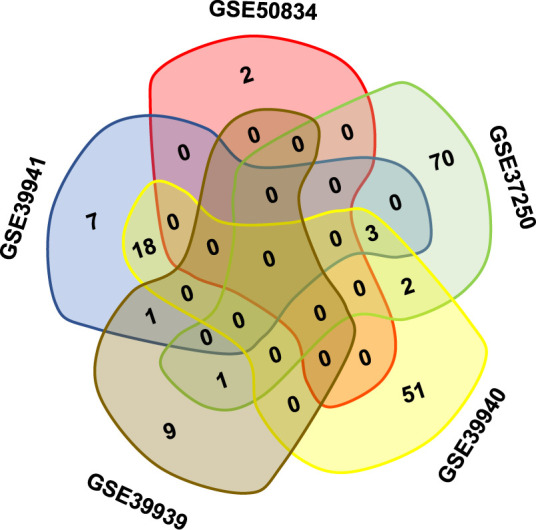
The overlapping DEGs in different studies on HIV positive patients with or without TB coinfection. Note that there was no gene present in all five datasets in the Venn diagram.

Gene expression responses to TB infection in patients who were HIV-positive were investigated by meta-analysis. Co-infection with other diseases was used as the control to eliminate responses that could be attributed to any other co-infections. The meta-analysis was conducted using publicly available microarray gene expression data implemented on a single platform (GPL10558 Illumina HumanHT-12 V4.0 expression chip). A total of five studies were selected for meta-analysis on 640 HIV-positive samples to identify DEGs (**Table 1**). All the samples were peripheral blood from patients including whole blood and PBMC. The samples were split into co-infections with TB and co-infections with other disease in each study (339 patients were co-infected with TB; 301 patients did not have TB). Following the traditional method, two-fold change was used as the criterion to screen DEGs from each study. The Venn diagram shows the overlapping DEGs in the five experiments; there were no DEGs present in all studies (**Figure 1**). The biggest overlap was between GSE33941 and GSE33940, which included 18 DEGs.

**Table 1:** Characteristics of transcriptome datasets used in this meta-analysis.

Accession	Title	Sample source	HIV + TB	HIV
GSE39941	Genome-wide transcriptional profiling of HIV positive and negative children with active tuberculosis, latent TB infection and other diseases	Whole Blood	68	93
GSE39940	Genome-wide transcriptional profiling of HIV positive and negative children with active tuberculosis, latent TB infection and other diseases from South Africa and Malawi	Whole Blood	41	66
GSE39939	Genome-wide transcriptional profiling of HIV positive and negative children with active tuberculosis, latent TB infection and other diseases from Kenya	Whole Blood	27	27
GSE50834	Gene expression analysis of PBMC from HIV and HIV/TB co-infected patients	PBMC	21	23
GSE37250	Genome-wide transcriptional profiling of HIV positive and negative adults with active tuberculosis, latent TB infection and other diseases	Whole blood	182	92

### Meta-Analysis of Differentially Expressed Genes in TB-HIV Coinfection

DEGs identified from the five studies were subjected to meta-analysis with the random effective model. A total of 11921 genes were tested and 293 DEGs had a P-value <0.0001 (**Table S1**). Of the 293 DEGs, 142 genes were up-regulated, while 151 genes were down-regulated. The top 20 most significantly up-regulated and down-regulated DEGs are shown in **Table 2**. Among them, *Guanylate Binding Protein 5* (*GBP5*) had the largest up-regulated TE-value=0.87 (P=1.57E-21), whereas gene only *RNase P catalytic subunit KIAA0391* had the largest down-regulated TE-value=-0.63 (P=3.51E-14). The top three significant DEGs were *LOC389386*, *Guanylate Binding Protein 4* (*GBP4*) and *GBP5*, with P<1.00E-20 in each case.

**Table 2:** The top 20 most significant DEGs after meta-analysis.

Genes	P value
***C9orf109***	4.2E−07
***SERPING1***	1.2E−17
***ANKRD22***	9.5E−06
***ATF3***	9.1E−10
***CYP4F22***	1.2E−05
***CDKN3***	8.3E−07
***SLC15A2***	1.2E−06
***ZWINT***	1.6E−06
***DONSON***	1.7E−06
***NCAPG***	4.2E−06
***SNORA63***	5E−06
***RRM2***	1.1E−05
***MND1***	1.9E−05
***C18orf56***	2.2E−05
***TIPIN***	4.7E−05

### Functional Category and Enrichment Analysis of Differentially Expressed Genes

**Figure 2:**
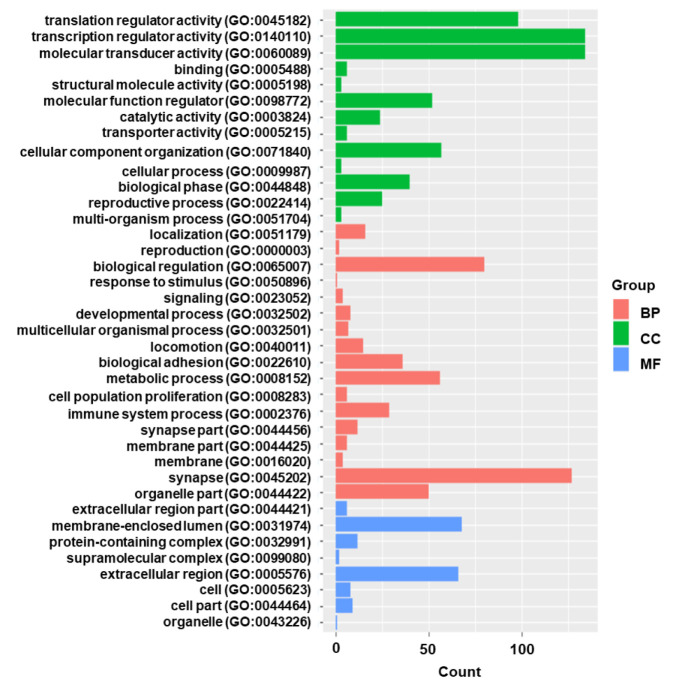
The gene functional classification of DEGs in HIV positive patients with TB compared with TB negative. BP, biological processes; CC, cellular compartment; MF, molecular function. Note that the most DEGs belonged to “catalytic activity” and “binding”.

Gene functional category analysis was performed to investigate the functions of the DEGs (**Figure 2**). According to their molecular function, most DEGs belonged to “catalytic activity” and “binding”. The DEGs were predominantly involved in biological processes such as “response to stimulus”, and “immune system process”.

**Figure 3:**
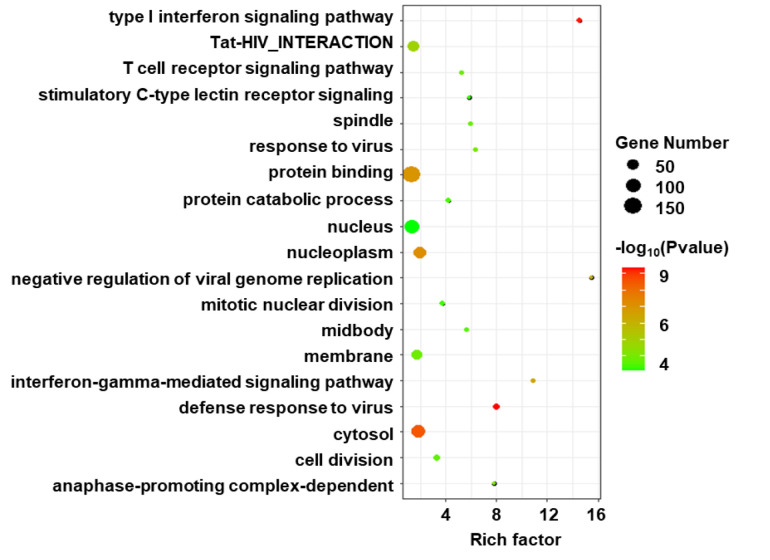
Gene ontology (GO) enrichment analysis of DEGs in HIV positive patients with TB compared with TB negative. The size of circle indicates the gene number; the color presents the log P-values. p < 0.05 and FDR < 0.01 were used as the threshold for pathway assignment. Note that the most DEGs were enriched in virus related pathway such as “defense response to virus” and “Type I interferon signaling”.

Gene ontology enrichment analysis was performed on the DEGs to further investigate the related biological pathways (**Figure 3**). The DEGs were most significantly enriched in “defense response to virus” (P-value=4.05E-10), followed by “Type I interferon signaling” (P-value=5.06E-10). Other enriched pathways included “negative regulation of viral genome replication”, “Tat-HIV_interaction”, “T cell receptor signaling” and “stimulatory C-type lectin receptor signaling” (P values <1.00E-03).

### Differentially Expressed Genes With Twofold Changes in Expression

**Figure 4:**
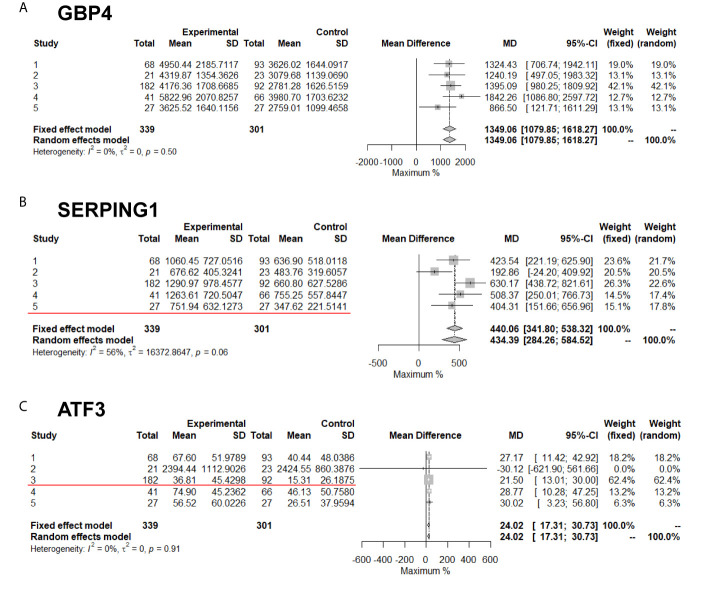
Meta-analysis of DEGs with or without twofold change among five studies. The Red line marked results are two-fold change. **(A)** GBP4; **(B)** SERPING1; **(C)** ATF3 genes' expression in meta-analysis.

Meta-analysis compares the difference values but not the ratio between experimental and control groups. Therefore, DEGs that overlapped between the meta-analysis and the traditional ratio method were subsequently identified (**Figure 4**). Up-regulated DEGs that had a two-fold change among the five studies included *C9orf109*, *SERPING1*, *ANKRD22*, *ATF3* and *CYP4F22*. Among them, *SERPING1* was the most significantly up-regulated with a P-value of 1.19E-17, followed by *ATF3* with P-value of 9.15E-10. Down-regulated DEGs that had a two-fold change among the five studies included *CDKN3*, *SLC15A2* and *ZWINT*. Among them, *CDKN3* was the most significantly down-regulated with a P-value of 8.32E-07.

### Candidate Gene Biomarkers for Tuberculosis in Patients Infected With Human Immunodeficiency Virus

**Figure 5:**
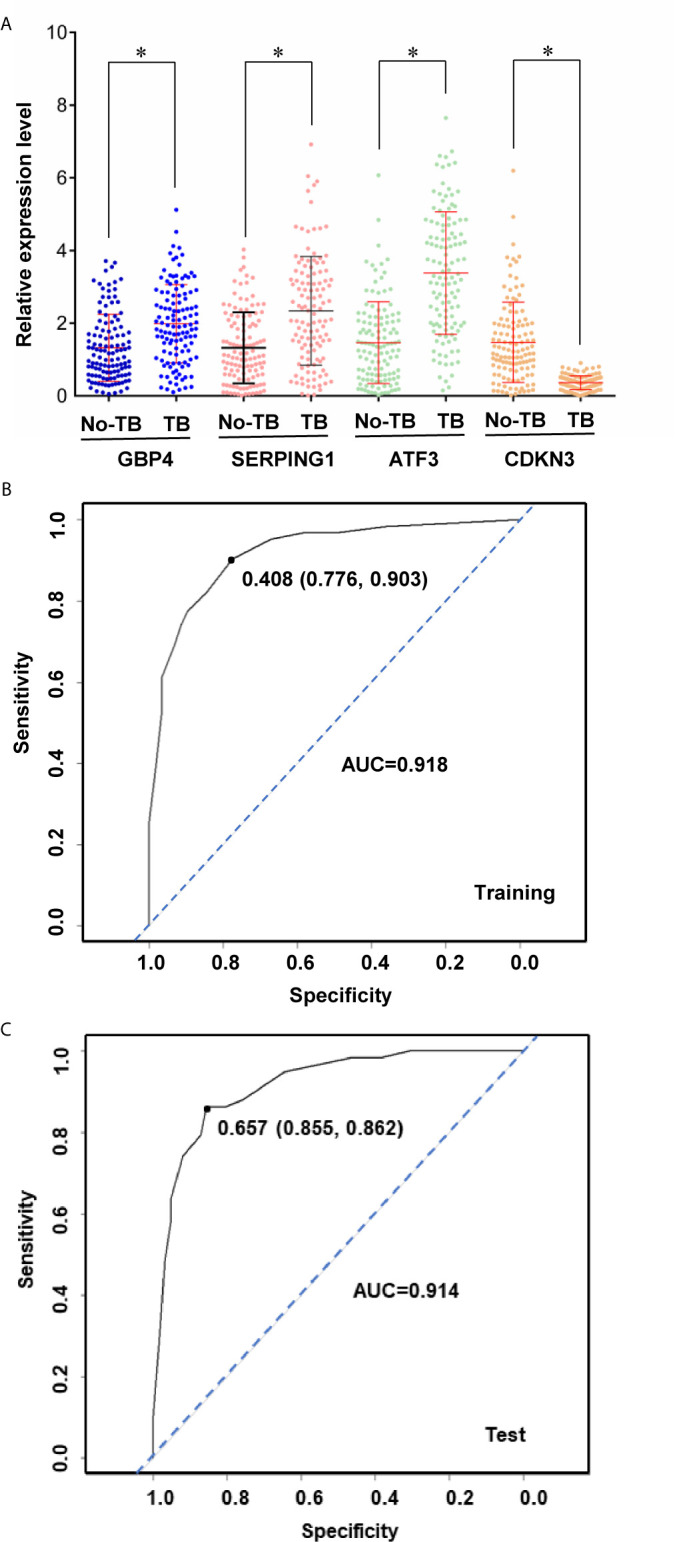
A biomarker panel to predict TB in patients infected with HIV. Relative expression level of genes **(A)** in HIV positive patients with or without TB coinfection. The expression data was then split into training and test sets to derive biomarker panels for Logistical regression model. Area under the receiver operating curve (AUC) is shown for the biomarker panels in training **(B)** and test **(C)** subjects. Stars indicate P < 0.05.

Based on the statistical significance of meta-analysis and the two-fold difference analysis, *GBP4*, *SERPING1*, *ATF3* and *CDKBN3* were selected as a biomarker panel to predict TB in patients infected with HIV. A clinical trial in patients with HIV with or without TB was performed to validate these biomarkers by measuring mRNA expression in PBMCs. First, relative expression levels of *GBP4*, *SERPING1*, *ATF3* and *CDKBN3* in experimental and control groups were determined by qRT-PCR. Expression of *GBP4*, *SERPING1* and *ATF3* was significantly up-regulated more than two-fold in the TB+HIV group compared with the control group, while *CDKN3* expression in the TB+HIV group was significantly reduced to half of that detected in the control group (**Figure 5A**). Next, the clinical trial was randomly split into modeling and validation groups. The modeling group was used to conduct a multivariable logistic regression analysis to establish an equation for predicting TB in patients with HIV by using the expression level of *GBP4*, *SERPING1*, *ATF3* and *CDKBN3* (**Figure 5B**). The validation group was then used to test this equation. This method generated an AUC >0.90 in both modeling and validation groups (**Figure 5C**). The optical sensitivity and specificity in the modeling group were 0.776 and 0.903, respectively, and 0.855 and 0.862, respectively, in the validation group.

## Discussion

Meta-analysis of five transcriptome studies with 11921 genes was conducted on 640 patients with HIV who were co-infected with or without TB. A total of 293 DEGs were identified, including 142 up-regulated and 151 down-regulated genes with statistical significance (P-value < 1E-4). Among these genes, 15 had at least a two-fold change in expression in one original transcriptome study. Comparing the results from each of the five transcriptome experiments, the traditional analysis method failed to provide multiple experimental repeats of DEGs; only 18 DEGs were found in two experimental repeats. The large genetic heterogeneity of clinical cases means that researchers doubt whether they can obtain critical biomarkers from clinical samples and statistical conclusions according to traditional analysis methods. However, meta-analysis provides convincing results by weighing the sample size and standard deviation of each experiment. Although the meta-analysis used in this study measures the difference rather than the ratio, some differences less than two-fold can provide supporting information for the statistics. A two-fold difference is often used as the benchmark in biological detection. Therefore, for potential biomarkers, priority was given in this study to the genes with a two-fold difference in at least one of the five transcriptome studies. In the further clinical sample validation, the expression level of these selected genes was detected by another technical means, qRT-PCR, and the statistically significant difference was in line with the expectation of meta-analysis. Meta-analysis of transcriptome datasets can better obtain disease markers with higher statistical standards from many clinical trials. This has been tested in practice in this HIV + TB study.

The meta-analysis of transcriptional profiles in this study identified DEGs enriched in some biological pathways of the host immune response, including IFNI response, T cell receptor and c-lectin receptor, and there is evidence that these pathways are related to TB infection. *Mycobacterium tuberculosis* can release cyclic-di-adenosine monophosphate (c-di-AMP), and stimulate release of cyclic guanosine monophosphate-adenosine monophosphate (cGAMP) in the host, both of which trigger the expression of type I IFN (Dey et al., 2017). *Tubercle DNA stimulated cyclic guanosine monophosphate (cGMP)-AMP synthase* (*CGA*) and enhanced type I interferon responses (Benmerzoug et al., 2019). Analysis of gene expression in the blood cells of susceptible mice infected with a clinical isolate of *Mycobacterium tuberculosis* revealed a regulation process similar to that of active tuberculosis in humans, including stimulation of type I interferon responses and activation of recruitment concentrated granulocytes (Moreira-Teixeira et al., 2020). In some experiments, a biomarker was developed to distinguish active tuberculosis patients using overexpression of interferon type I-induced gene signature (Singhania et al., 2018). The T-cell receptor is also associated with tuberculosis infection. Clinical investigation showed that the signal pathway of the T-cell receptor interleukin-2 (IL-2) inducible T-cell kinase (ITK) was altered in patients with active tuberculosis (Huang et al., 2019). Moreover, there is some evidence that the c-lectin receptor pathway is closely related to tuberculosis infection. C-type lectin receptors, like Toll-like receptors and NOD-like receptors, participate in the immune response to *Mycobacterium tuberculosis* infection (Mishra et al., 2017). C-type lectin receptors, after binding with the ligand, affect the immune response process by regulating the type I interferon signal in dendritic cells (Troegeler et al., 2017). From the perspective of biological function, the results of the meta-analysis of the transcriptional profiles of DEGs in our study are consistent, to some extent, with the host immune response to *Mycobacterium tuberculosis*.

Among the 293 DEGs obtained from the meta-analysis, the four most statistically significant genes were selected as a combination to predict a combined TB infection in patients infected HIV. In addition to the statistical significance of *GBP4*, *SERPING1*, *ATF3* and *CDKBN3* in our analysis, many studies have also shown that these genes are excellent markers of tuberculosis infection. According to genome-wide expression profiling, the interferon type I response pathway is an important pathway in active TB, and the key response genes of this pathway, *IL15RA*, *UBE2L6* and *GBP4*, and their cascade signals can be used to monitor active TB (Ottenhoff et al., 2012). In addition, many groups of TB patients showed significant up-regulation in expression of endogenous complement regulatory factor C1 inhibitor encoded by *SERPING1*. Moreover, in the mouse model, *ATF3* was expressed in macrophages infected with *Mycobacterium tuberculosis (* Seimon et al., 2010). *ATF3* regulates the immune response of macrophages to *Mycobacterium tuberculosis* infection through the formation of inflammatory genes and liposomes (Kumar et al., 2020). The combination of these four genes identified in our meta-analysis study predicted TB with a higher AUC in patients with HIV, and therefore form a valuable biomarker panel.

In conclusion, meta-analysis is an effective method to integrate the results of multiple transcriptional profiling studies. In this study, a panel of reliable marker genes were identified and used to construct a model through multiple logistic regression analysis that was able to effectively predict TB co-infection in patients infected with HIV.

## Data Availability Statement

The datasets presented in this study can be found in online repositories. The names of the repository/repositories and accession number(s) can be found in the article/**Supplementary Material**.

## Ethics Statement

The studies involving human participants were reviewed and approved by the Ethical Committee of Mengchao Hepatobiliary Hospital of Fujian Medical University. The patients/participants provided their written informed consent to participate in this study.

## Author Contributions

YC, QW, and SL designed the project, performed the meta-analysis, and wrote the draft. JLa, JLi, and WA collected the transcriptome datasets and processed the raw data. XH and HY designed the project, supervised the experiment, and wrote the manuscript. All authors contributed to the article and approved the submitted version.

## Funding

This study was supported by the Clinical Medicine Center Construction Program of Fuzhou, Fujian, P.R.C (2018080306), Key Clinical Specialty Discipline Construction Program of Fujian, P.R.C, and Joint Research Project of Health and Education in Fujian Province (2019-WJ-15). Fujian Chinese Traditional Medicine University research funding (2017ZX10205502-003).

## Conflict of Interest

The authors declare that the research was conducted in the absence of any commercial or financial relationships that could be construed as a potential conflict of interest.
